# Automated GMP-Compliant Production of [^68^Ga]Ga-DO3A-Tuna-2 for PET Microdosing Studies of the Glucagon Receptor in Humans

**DOI:** 10.3390/ph13080176

**Published:** 2020-07-31

**Authors:** Michael Wagner, Johan G. Doverfjord, Joachim Tillner, Gunnar Antoni, Torsten Haack, Martin Bossart, Iina Laitinen, Lars Johansson, Stefan Pierrou, Olof Eriksson, Irina Velikyan

**Affiliations:** 1 R&D Research Platform, Integrated Drug Discovery, Sanofi, 65929 Frankfurt, Germany; Torsten.Haack@sanofi.com (T.H.); martin.bossart@sanofi.com (M.B.); 2 PET Center, Center for Medical Imaging, Uppsala University Hospital, 751 85 Uppsala, Sweden; johan.gustav.doverfjord@akademiska.se; 3 Translational Medicine, Sanofi, 65929 Frankfurt, Germany; joachim.tillner@sanofi.com; 4 Department of Medicinal Chemistry, Uppsala University, 751 23 Uppsala, Sweden; gunnar.antoni@akademiska.se; 5 Global Imaging, Sanofi, 65929 Frankfurt, Germany; iina.laitinen@antarosmedical.com; 6 Antaros Medical AB, 431 83 Mölndal, Sweden; lars.johansson@antarosmedical.com (L.J.); stefan.pierrou@antarosmedical.com (S.P.); olof.eriksson@ilk.uu.se (O.E.); 7 Science for Life Laboratory, Department of Medicinal Chemistry, Uppsala University, 751 23 Uppsala, Sweden

**Keywords:** glucagon receptor, diabetes, GMP, Gallium-68, automation, radiopharmaceuticals

## Abstract

**Introduction**: [^68^Ga]Ga-DO3A-VS-Cys^40^-Tuna-2 (previously published as [^68^Ga]Ga-DO3A-VS-Cys^40^-S01-GCG) has shown high-affinity specific binding to the glucagon receptor (GCGR) in vitro and in vivo in rats and non-human primates in our previous studies, confirming the suitability of the tracer for drug development applications in humans. The manufacturing process of [^68^Ga]Ga-DO3A-VS-Cys^40^-Tuna-2 was automated for clinical use to meet the radiation safety and good manufacturing practice (GMP) requirements. **Methods:** The automated synthesis platform (Modular-Lab PharmTrace, Eckert & Ziegler, Eurotope, Germany), disposable cassettes for ^68^Ga-labeling, and pharmaceutical-grade ^68^Ge/^68^Ga generator (GalliaPharm^®^) used in the study were purchased from Eckert & Ziegler. The parameters such as time, temperature, precursor concentration, radical scavenger, buffer concentration, and pH, as well as product purification step, were investigated and optimized. Process optimization was conducted with regard to product quality and quantity, as well as process reproducibility. The active pharmaceutical ingredient starting material DO3A-VS-Cys^40^-Tuna-2 (GMP-grade) was provided by Sanofi Aventis. **Results:** The reproducible and GMP-compliant automated production of [^68^Ga]Ga-DO3A-VS-Cys^40^-Tuna-2 with on-line documentation was developed. The non-decay-corrected radiochemical yield was 45.2 ± 2.5% (*n* = 3, process validation) at the end of the synthesis with a labeling synthesis duration of 38 min and a quality controlincluding release procedure of 20 min. The radiochemical purity of the product was 98.9 ± 0.6% (*n* = 17) with the total amount of the peptide in the preparation of 48 ± 2 µg (*n* = 3, process validation). Radionuclidic purity, sterility, endotoxin content, residual solvent content, and sterile filter integrity tests met the acceptance criteria. The product was stable at ambient temperature for at least 2 h. **Conclusion:** The fully automated GMP-compliant manufacturing process was developed and thoroughly validated. The resulting [^68^Ga]Ga-DO3A-VS-Cys^40^-Tuna-2 was used in a clinical study for accurate quantification of GCGR occupancy by a dual anti-diabetic drug in vivo in humans.

## 1. Introduction

Drug development is a process of high expense and high failure rate [1]. Considerable improvement in drug development has occurred during recent years by shifting from empirical approaches to more mechanistic and predictive ones based on the achievements of molecular biology and imaging. Another critical aspect of the success is the realization of the microdosing concept that allows us to overcome the regulatory hurdle of evaluating imaging agents by standards relevant to therapeutic agents. Microdosing, particularly positron emission tomography (PET) microdosing [2,3,4], is recognized by the EMEA and FDA, and the Exploratory Investigational New Drug (eIND) guidelines reduce the demand on toxicity studies and respective cost burden [5,6]. This is possible because of the high sensitivity of PET and, consequently, the use of non-pharmacological radiopharmaceutical doses of pico-/nanomoles (nanograms-micrograms).

PET-microdosing simplifies the initiation of clinical studies and accelerates the process of selection or rejection of a drug candidate based on pharmacokinetic studies in vivo in humans at a very early stage defined as a Phase 0 study. It reduces the overall costs of drug development drastically. The prediction of drug behavior in humans based on animal studies is not sufficiently accurate, and human trials remain the ultimate means for the stratification of candidate drugs. An important aspect of PET is valuable information on the target distribution, quantification, and occupancy throughout the body obtained non-invasively in vivo. It is also worth mentioning that adverse reactions to PET radiopharmaceuticals are extremely rare and with no serious or life-threatening events [7].

There is an unmet medical need for the treatment of type 2 diabetes (T2D) and obesity given a constantly increasing population affected by T2D and/or obesity worldwide [8,9]. A constant supply of new drugs is necessary. The glucagon receptor (GCGR) has been considered a potential target for T2D management since the 1990s. It is expressed in the liver and kidney, with lower amounts found in the heart, spleen, pancreas, adrenals, and gastrointestinal tract. Agonism at the GCGR is associated with several important metabolic processes, including glucose homeostasis (increase in blood glucose through, for example, glycogenolysis in liver) but also decreased food intake and increased expenditure [10]. A therapeutic drug targeting both GCGR and glucagon-like peptide-1 receptors (GLP1R) is anticipated to provide glucose control combined with a clinically meaningful weight reduction [11,12,13]. The development of such novel dual anti-diabetic therapeutic drugs would benefit from the employment of PET that would enable the in vivo investigation of GCGR/GLP1R engagement by the therapeutic agent. Furthermore, monitoring the disease status during therapeutic intervention would allow adjustment of the treatment very early in the process if the drug becomes ineffective over time. Prediction of the drug efficacy on the individual basis is another value that such a method would offer.

Both preclinical and clinical studies have demonstrated the strong potential of GCG/GLP1 receptor agonists for therapeutic effects such as weight loss and the stabilization of glycemic level [11,12,13,14]. An early target occupancy investigation using PET in combination with radiolabeled ligands for each of the intended target receptors would play an important role in the acceleration of the development process. We have recently published promising results on the specific binding of a novel ^68^Ga-labeled GCGR ligand, [^68^Ga]Ga-DO3A-S01-GCG (herein named [^68^Ga]Ga-DO3A-VS-Cys^40^-Tuna-2), preclinically in vitro and in vivo in rats and non-human primates [15,16]. The ^68^Ga-labeling of the ligand comprising DOTA (1,4,7,10-tetraazacyclododecane-1,4,7,10-tetraacetic acid) bifunctional chelator was developed and conducted manually in order to assure the highest possible specific activity [MBq/µg] (molar activity, [MBq/nmol]) requested for the investigation of target occupancy by anti-diabetic peptides in animals. This study was devoted to the development and validation of an automated and good manufacturing practice (GMP)-compliant production of [^68^Ga]Ga-DO3A-VS-Cys^40^-Tuna-2 for human use.

## 2. Material and Methods

### 2.1. Facilities, Equipment, and Materials

Synthesis of DO3A-VS-Cys^40^-Tuna-2 was published earlier [15]. The peptide constitutes 39 amino acid residues (TzaD-SerQGTFTSDYSKQLDE*QRAK*EFIEWLLATGPESGAPPPS) and is coupled to DOTA via a bifunctional chelator via cysteine amino acid residue at position 40 and a vinyl sulfone linker. The active pharmaceutical ingredient starting material, designated DO3A-VS-Cys^40^-Tuna-2 (GMP-grade drug product), was synthetically produced and provided by Sanofi (Frankfurt, Germany) and its collaboration partners. The manufacturer followed GMP guidelines. Safety, pharmacology, and toxicology studies in animals revealed no DO3A-VS-Cys^40^-Tuna-2 related toxicity. Certificates of compliance and certificates of analysis were obtained from the manufacturer. The product was sterile, non-pyrogenic, and without bacteriostatic preservatives. Purchased chemicals were used without further purification: HCl (ultrapure, Merck, Darmstadt, Germany), sodium acetate buffer (pH 4.6, Sigma-Aldrich, St. Louis, MO, USA), sterile water (Fresenius Kabi, Uppsala, Sweden), sterile saline (0.9%, Apoteket AB, Uppsala, Sweden), NaOH (10 M, Sigma-Aldrich, St. Louis, MO, USA), ethanol (APL, Apotek Produktion & Laboratorier, Uppsala, Sweden), water (Fluka, TraceSelect, Uppsala, Sweden), trifluoroacetic acid (Merck, St. Louis, MO, USA).

The aseptic production was conducted in a GMP grade A workstation (unidirectional laminar airflow workbench (LAFW)) situated in a cleanroom with GMP grade B air quality. The ^68^Ge/^68^Ga generator (GalliaPharm^®^, Eckert & Ziegler, Berlin, Germany) and Modular PharmLab labeling synthesis platform (Eckert & Ziegler Eurotope, Berlin, Germany) were placed in the LAFW. A high-performance liquid chromatography system (LaChrom, Hitachi, VWR) consisting of an L-2130 pump, UV detector (L-2400), and radiation flow detector (Bioscan) coupled in series was used for product quality control. Separation of the analytes was accomplished using an analytical column with a stationary reversed phase (C-4; Vydac-C4; 50 × 4.6 mm; particle size: 3 µm). The conditions were as follows: A = 10 mM trifluoroacetic acid in water; B = 100% acetonitrile (MeCN) with 10 mM TFA with UV-detection at 220 nm; linear gradient elution: 0 min at 24% B, 0–8 min from 24 to 45% B, 8–10 min at 45% B, 10–15 min at 24% B; flow rate was 2.0 mL/min. Data acquisition and handling were performed using the EZChrom Elite Software Package (version 1.3).

The starting material, ^68^Ga (t_1/2_ = 68 min, β^+^ = 89%, and electron capture= 11%), used for the production was obtained from the pharmaceutical-grade ^68^Ge/^68^Ga-generator (1850 MBq, GalliaPharm^®^) by elution with 0.1 M hydrochloric acid. The amount of detected metal impurities as provided by the manufacturer was less than the defined limit in the European Pharmacopeia monograph [17]. The appearance of the ^68^Ga eluate was clear and colorless. The ^68^Ge breakthrough in the eluate as a percentage of the eluted ^68^Ga radioactivity was calculated by measuring aliquots of the generator eluate and counting the radioactivity content using an ionization chamber with a NaI(Tl) scintillation detector immediately after elution and in the well-type NaI(Tl) scintillation counter after 48 h post-elution.

### 2.2. Production and Quality Control of [^68^Ga]Ga-DO3A-VS-Cys^40^-Tuna-2

The production was conducted using a commercial fully automated platform for labeling synthesis (Modular-Lab PharmTrace (Eckert & Ziegler, Eurotope, Germany)) with a disposable cassette system (C4-Ga68-PP). The running program was modified to accommodate the specifics of the synthesis algorithm. The process included labeling synthesis, product purification, formulation, and sterile filtration, as well as a sterile filter integrity test. Such parameters as reaction time, temperature, and radioactivity were monitored in real time. The generator elution profile was optimized to provide a top fraction of approximately 3.2 mL with the highest radioactivity content. A solid phase extraction (SPE) cartridge (tC2, Waters, Solna, Sweden) was used for the product purification. The cassette was loaded with an SPE cartridge, reaction mixture containing the reaction buffer (1 M sodium acetate buffer, 1 M NaOH, dihydroxybenzoic acid, and ethanol in total volume of 0.9 mL, pH = 7.4 ± 0.2), and precursor (15–20 µM, 0.9 mL, DO3A-VS-Cys^40^-Tuna-2) that was added to the reactor positioned in the heating block. The reaction mixture was heated in a conventional heating block for 15 min at 75 °C. Three milliliters of sterile saline was added to the reaction mixture in order to cool it down prior to loading onto the SPE cartridge for the purification. After loading, another 2 mL of saline was passed through the cartridge to wash it from possible residual free cationic ^68^Ga(III). Thereafter, the product was eluted with 1 mL of 50% ethanol. For the product formulation, 6 mL of saline was added to assure a content of ethanol of less than 10% in the final product. The solution was then passed through a 0.22 µm sterile filter into a sterile 27 mL capped glass bottle. The integrity of the sterile filter was controlled in-line on the same platform using a separate program. Prior to the patient administration, the quality control determining the identity, radiochemical purity, peptide concentration, and pH was performed, and the product was released for clinical use by an independent certified operator or qualified person. The total radioactivity of the product was then measured by clinical staff in a dose calibrator prior to the administration. The duration of the synthesis program was 38 min and the total duration of the production including quality control was approximately 60 min.

Quality control on the chemical purity, radiochemical purity, and determination of the peptide concentration was performed using UV-Radio-HPLC. The development and validation of the method were conducted using two different reversed-phase columns Vydac-C4 and Aeris-C4 with mobile phases of 0.1% TFA in water as the A mobile phase and 0.1% TFA in acetonitrile as the B mobile phase. The gradient of B increased from 25% to 40% within 30 min for Aeris-4 and B increased from 24 to 45% within 8 min, followed by 2 min of isocratic elution. The recovery of radioactivity from the analytical column was investigated, in order to confirm that no radioactive product or impurities were left on the column, by collecting HPLC effluent with and without an analytical column and measuring the radioactivity. The tests were performed for both the product ([^68^Ga]Ga-DO3A-VS-Cys^40^-Tuna-2) and free ^68^Ga(III). Specificity, linearity, and precision as repeatability were validated for both UV- and radio-detectors.

Three successful sequential productions were performed for the manufacturing validation. A fraction of pre-determined volume was kept for the subsequent determination of ^68^Ge content and solvent residual content. The stability of the product at room temperature was monitored by UV-radio-HPLC for 2 h.

### 2.3. Clinical Examination

[^68^Ga]Ga-DO3A-VS-Cys^40^-Tuna-2 PET/CT was used for assessing the hepatic GCGR receptor density and availability within a phase Ib, open-label study investigating the receptor occupancy of a dual GLP1/GCG agonist (ClinicalTrials.gov: NCT03350191). The results of the full receptor occupancy study, as well as the human biodistribution and dosimetry of [^68^Ga]Ga-DO3A-VS-Cys^40^-Tuna-2, will be reported separately. The study was approved by the Swedish Medical Products Agency and the regional ethical review board (Dnr 2017/395; EudraCT nr. 2017-001789-23).

Briefly, individuals with T2D were enrolled in the study and signed informed consent. They (*n* = 13) were placed in the supine position with the liver in the 20 cm axial PET field of view of a Discovery MI PET/CT scanner (GE Healthcare). [^68^Ga]Ga-DO3A-VS-Cys^40^-Tuna-2 (target dose 0.5 MBq/kg, <0.2 µg/kg peptide) was administered intravenously as a bolus followed by a 60 min dynamic PET acquisition in list-mode. The PET list mode data were reconstructed into 30 frames (12 × 10, 6 × 30, 5 × 120, 5 × 300, 2 × 600 s) using an iterative VPFX-S algorithm (3 iterations, 3 subsets, matrix 256 × 256, *Z*-axis post-filter 3 mm) with all relevant corrections performed.

The first 10 individuals exposed to [^68^Ga]Ga-DO3A-VS-Cys^40^-Tuna-2 were monitored by a dedicated physician during the examination according to the local first-in-man monitoring protocol, which includes continuous 12-lead ECG, blood pressure, inspection of injection site, and inspection of the general condition before and after administration, as well as a 24 h follow-up of ECG and adverse events.

## 3. Results

### 3.1. Production of [^68^Ga]Ga-DO3A-VS-Cys^40^-Tuna-2

**Figure 1:**
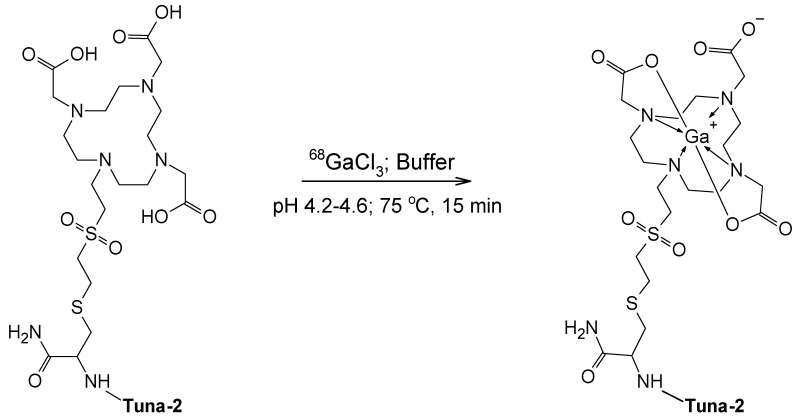
Schematic presentation of labeling synthesis of [^68^Ga]Ga-DO3A-VS-Cys^40^-Tuna-2. Tuna-2 constitutes 39 amino acid residues (TzaD-SerQGTFTSDYSKQLDE*QRAK*EFIEWLLATGPESGAPPPS, wherein the asterisk denotes a lactam bridge) and is coupled to DOTA via a bifunctional chelator via cysteine amino acid residue at position 40 and a vinyl sulfone linker.

The schematic presentation of the labeling synthesis of [^68^Ga]Ga-DO3A-VS-Cys^40^-Tuna-2 with structural details is given in Figure 1.

**Figure 2:**
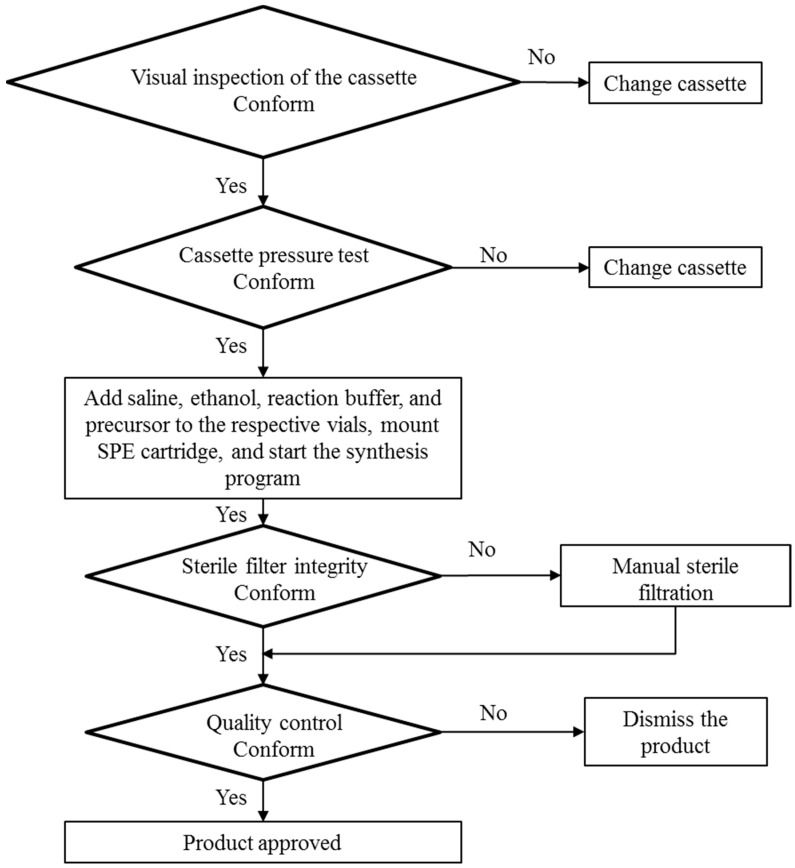
Flowchart of the production of [^68^Ga]Ga-DO3A-VS-Cys^40^-Tuna-2 on the Modular-Lab PharmTrace.

**Figure 3:**
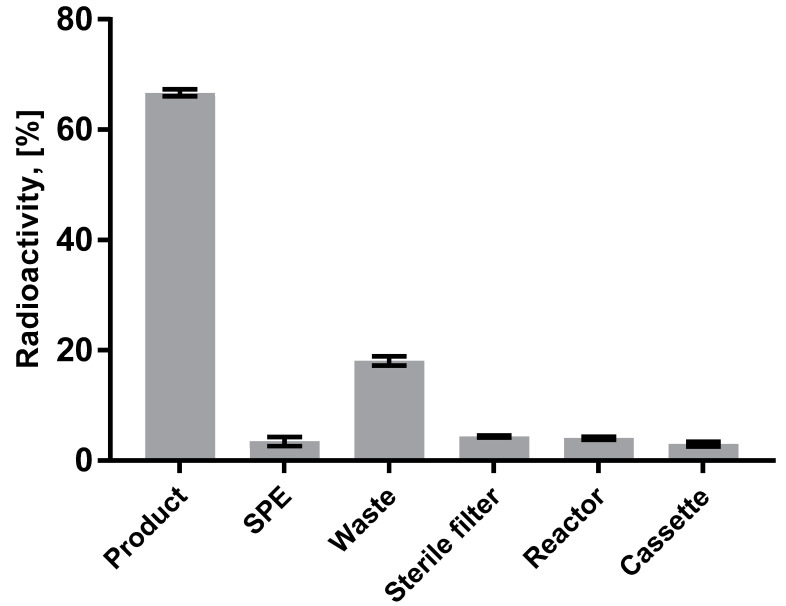
Distribution of the radioactivity on the cassette presented as a fraction (%) of the total radioactivity entering the cassette from the generator. Data are presented as mean ± SD (*n* = 4).

**Figure 4:**
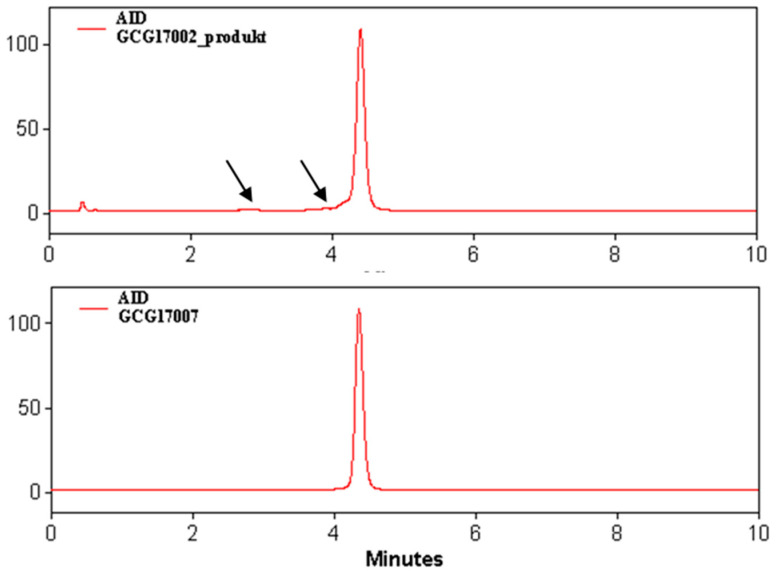
Radio-chromatograms of the formulated product resulting from the production without (upper panel) and with (lower panel) addition of radical scavengers such as ethanol and dihydroxybenzoic acid. The traces of presumably oxidative radiolysis products (black arrows, upper panel) completely disappeared (lower panel) in the presence of ethanol and dihydroxybenzoic acid.

The flowchart (Figure 2, Modular-Lab PharmTrace) of the fully automated production of [^68^Ga]Ga-DO3A-VS-Cys^40^-Tuna-2 highlights the critical steps. Figure 3 presents the distribution of the radioactivity in the cassette for the three validation productions. The radioactivity fraction of the adsorption on the cassette manifold was calculated as the difference between the measured components and the initially expected total amount of the radioactivity entering the cassette from the ^68^Ge/^68^Ga generator. The low relative standard deviation (RSD) indicated reliability of the production process. The waste fraction consists of the generator elution waste fractions (1st and 3rd) and waste fraction of the SPE purification of the crude product. The waste fraction RSD of 17.8% indicates high reproducibility of the labeling synthesis. The elution profile of [^68^Ga]GaCl_3_ from the generator was optimized to yield over 90% in the top fraction of 3.0–3.5 mL (2nd fraction). The formation of by-products of 2–5% was fully suppressed by addition of ethanol and dihydroxybenzoic acid (Figure 4).

**Figure 5:**
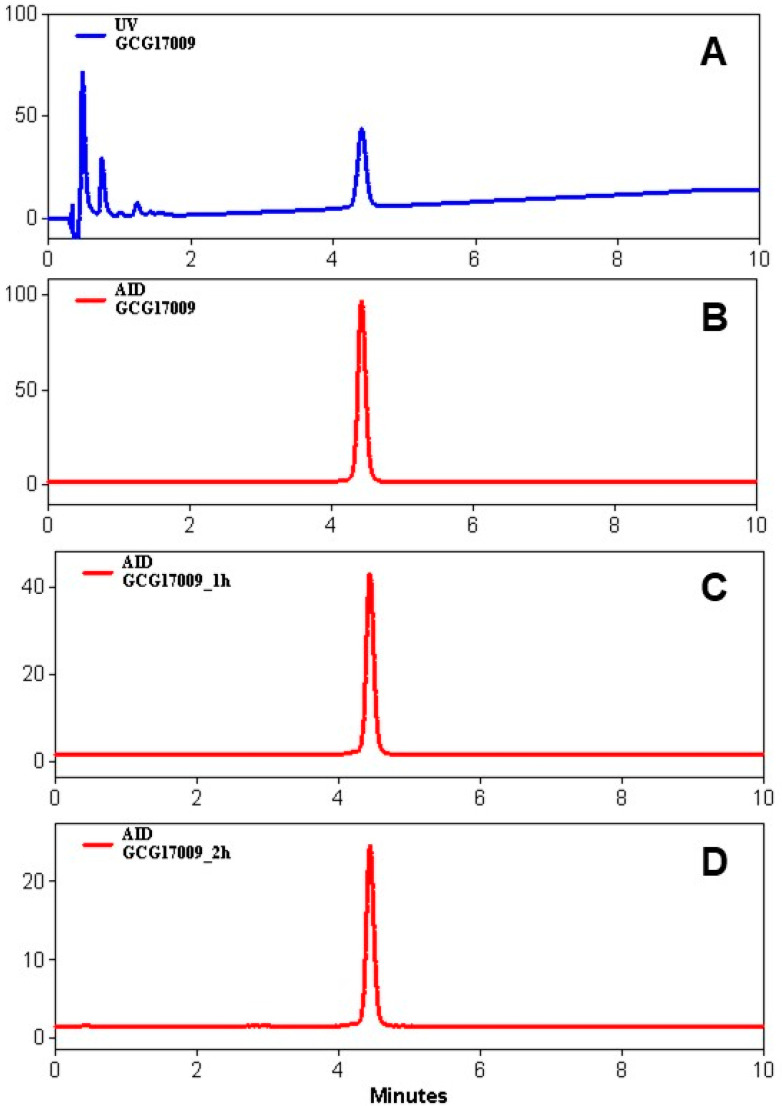
Stability of the formulated product at room temperature: (**A**) UV-trace; (**B**–**D**) radioactivity trace after production (**B**), 1 h (**C**), and 2 h (**D**) post-production, showing a radiochemical purity (RCP) of over 98%.

The concentration (0.3 M) and pH (pH = 7.4 ± 0.2) of the acetate buffer adjusted by addition of sodium hydroxide (30 µL, 10 M) was optimized in order to compensate for the addition of acidic generator eluate solution and provide favorable and robust pH. The product purification step was optimized by testing reversed-phase SPE cartridges with C2, C8, and C18 stationary phases from Waters. The product retention was over 99% for all cartridges, while a recovery of over 95% was best for the C2 variant. The product was diluted with sterile sodium chloride (0.9%) on the formulation step with a final total volume of 5.78 ± 0.13 mL (*n* = 3, production validation runs) so that the final ethanol concentration would be <10%. The radiochemical yield of crude product was over 90% for the reaction carried out at 75 °C for 15 min. The developed method was GMP-compliant, reliable, and reproducible with a radiochemical purity (RCP) of 98.9 ± 0.6 (*n* = 17). The product was stable at ambient temperature for at least 2 h with radiochemical purity directly after the production of 99.3 ± 0.5 and 2 h postproduction of 97.9 ± 0.9% for production validation runs (*n* = 3) (Table 1, Figure 5). The molar activity determined by the ratio of the radioactivity measured in an ionization chamber to the total amount of the peptide determined by UV-HPLC was 36.5 ± 1.6 MBq/nmol (*n* = 3, production validation runs) given the radioactivity amount entering the synthesis was estimated as 850 MBq. The non-decay-corrected radiochemical yield was 45.2 ± 2.5% (*n* = 3, production validation runs).

The final product [^68^Ga]Ga-DO3A-VS-Cys^40^-Tuna-2 was passed through a 0.22 µm sterile filter disk in-line. The sterile filter integrity test was conducted automatically using another program sequence. A sample of the product was kept for subsequent determination of ^68^Ge content and resulted in less than 0.00002%. Tests performed on the finished product, specifications, and results are summarized in Table 2.

The generator (GalliaPharm^®^, Eckert & Ziegler) qualification was published in detail previously [18].

### 3.2. Quality Control

Radiochemical purity, chemical purity, and peptide concentration in the final product were determined by UV-radio-HPLC. The validation of the UV-radio-HPLC analysis method was conducted using two different reversed-phase columns (Vydac-C4 and Aeris-C4), as well as two different elution systems with different mobile phases and elution gradients. The results demonstrated high reliability and reproducibility in terms of specificity, linearity, precision, and repeatability with respect to both UV- and radio-detectors, as well as radioactivity recovery from the HPLC columns (Table 3).

**Figure 6:**
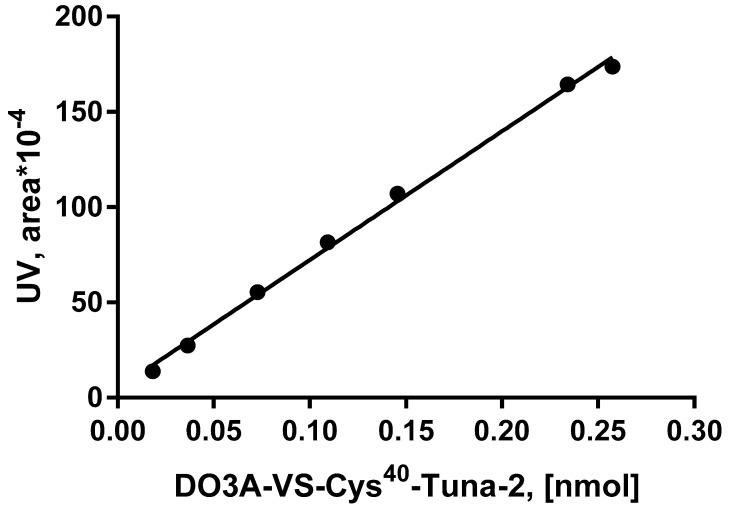
UV-calibration plot (*R^2^* = 0.9972) of DO3A-VS-Cys^40^-Tuna-2 for the HPLC quality control process validation and determination of the peptide concentration in the product.

The determination of the peptide content in the radiolabeled product was conducted based on the calibration of the UV-signal (Pearson correlation coefficient (*R^2^*) of >0.9972) (Figure 6) in the range of peptide concentrations corresponding to that expected for the formulated product. The recovery of radioactivity from the HPLC column was found to be 99% and 97%, respectively, for the final product and the product spiked with [^68^Ga]GaCl_3_, assuring the adequate interpretation of the analysis results. The quality control procedure included a system suitability test (UV-HPLC) conducting analysis using a standard reference of known concentration. The results were used also for one-point calibration to confirm the validity of the calibration plot and cross-controlling the peptide concentration in the formulated product.

### 3.3. Clinical Examination

**Figure 7:**
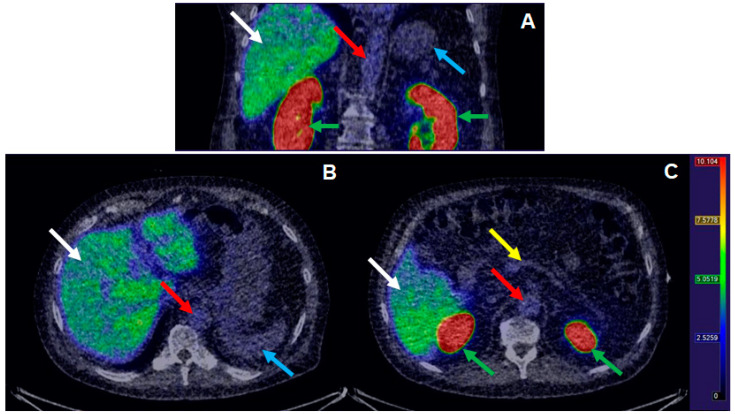
Biodistribution of [^68^Ga]Ga-DO3A-Tuna-2 in selected tissues after IV administration in patient (39 MBq, 0.45 MBq/kg, 10.2 µg peptide, 0.12 µg/kg peptide). Representative coronal (**A**) and transaxial (**B**,**C**), images of the abdomen. Images are summations of 20–60 min after administration and normalized to SUV 10. White arrows indicate liver, red aorta, blue spleen, yellow pancreas, and green kidney.

[^68^Ga]Ga-DO3A-VS-Cys^40^-Tuna-2 was successfully administered to perform, in total, *n* = 21 PET examinations during the course of the clinical study. On average, 0.47 ± 0.04 MBq/kg [^68^Ga]Ga-DO3A-VS-Cys^40^-Tuna-2 was administered, corresponding to 0.12 ± 0.06 µg/kg. The target peptide dose (<0.2 µg/kg) was met in all examinations except one (0.33 µg/kg). No adverse events deemed directly associated with the administration of [^68^Ga]Ga-DO3A-VS-Cys^40^-Tuna-2 were recorded during the first-in-man monitoring procedure. [^68^Ga]Ga-DO3A-VS-Cys^40^-Tuna-2 demonstrated rapid plasma kinetics and strong binding in the liver during baseline examinations (Figure 7). It also exhibited trapping in the kidney cortex, indicating mainly renal excretion, as previously demonstrated in several animal models.

## 4. Discussion

PET allows non-invasive, whole body, accurate quantification on the molecular level with high sensitivity and specificity. It provides the possibility for accurate monitoring of the receptor expression, drug function related to the same target, and follow up of disease progression, as well as therapy response monitoring. To facilitate drug development and allow the successful drug stratification early in development, PET plays an important role especially in the context of microdosing and Phase 0 clinical trials. In this study, an automated manufacturing of [^68^Ga]Ga-DO3A-VS-Cys^40^-Tuna-2 was developed for the validation of a dual receptor targeting anti-diabetic peptide in terms of receptor occupancy quantification in vivo in humans.

[^68^Ga]Ga-DO3A-VS-Cys^40^-Tuna-2 (previously referred to as [^68^Ga]Ga-DO3A-S01-GCG) is a novel peptide-based imaging agent specifically targeting GCGR present on hepatocytes [15,19]. It is based on a peptide of ca 5 kDa coupled to a DOTA bifunctional chelator via a cysteine residue and a divinyl sulfone linker resulting after the ^68^Ga-labeling in a neutral complex moiety (Figure 1) [15]. It demonstrated promising results in vitro and ex vivo in cells and frozen tissue cross-sections, in vivo in rats and non-human primates (NHP) [15,19]. Labeling synthesis was conducted manually for the preclinical studies. However, clinical application poses stricter demands on the production robustness and GMP compliance, product quality, risks of failure, and exposure of the operator to the radiation. The outline of the clinical study was complex, requiring repeated productions within a tight time frame, and, thus, the frequency and success rate of the automated production were crucial.

### 4.1. Automated Production of DO3A-VS-Cys^40^-Tuna-2

In general, the automated production of a radiopharmaceutical compared to the manual production improves manufacturing robustness, enables reduction in radiation exposure to the operator, allows on-line recording and documentation for the traceability, and simplifies the transfer of the radiopharmaceutical production process to other sites [20]. The Modular-Lab PharmTrace platform is based on a disposable cassette concept and provides labeling methods using either fractionation or pre-concentration of the generator eluate. It excludes the risk of cross-contamination and allows a high synthesis frequency, which was crucial for the clinical study. The automated method development was based on the manual labeling procedure [15] and on the experience of the automated production of [^68^Ga]Ga-DO3A-VS-Cys^40^-Exendin-4 [18]. Special attention was given to the optimization of the synthesis time and precursor peptide concentration influencing the radiochemical yield and, consequently, the molar activity value, as the clinical study limited the amount of the injected peptide mass to 0.2 µg/kg body weight based on the preclinical study in non-human primates, elucidating the effect of administered peptide mass on the GCGR occupancy [19].

DO3A-VS-Cys^40^-Exendin-4 was more sensitive to the acidity of the reaction mixture and required pre-concentration of the generator eluate in order to avoid the addition of a high volume of hydrochloric acid solution [18], while DO3A-VS-Cys^40^-Tuna-2 demonstrated tolerance toward the addition of the generator eluate of up to 3.2 mL. The use of the fractionation method shortened the synthesis time by about 10 min, thus enhancing the molar activity of the product.

Parameters such as temperature and buffer type (acetate buffer), as well as purification step, were similar to those used previously [15,18] and did not require further optimization, while pH and radical scavenger type and concentration, as well as precursor concentration, had to be optimized. The pH of the buffer was adjusted to 7.4 ± 0.2 in order to compensate for the use of the fractionation method and, thus, total amount of the acidic generator eluate. The resulting reaction mixture pH was 4.2–4.6.

The amount of the precursor used in the labeling synthesis was 15 nmoles in order to meet the balance between the reaction efficiency, molar activity, and final concentration of the peptide, allowing the HPLC determination of the concentration. This amount corresponded to 3 µM of reaction solution and provided a non-decay-corrected radiochemical yield of 45.2 ± 2.5% (*n* = 3, production validation), wherein the relative standard deviation of 5.4% indicates the solid repeatability of the process.

The purification of the final product was performed on the SPE cartridge tC2 using 50% ethanol for the product recovery that allowed, on the one hand, the lowest possible formulation dilution and, on the other hand, the highest possible peptide concentration, permitting the accurate determination of the latter by UV-HPLC. The distribution of the radioactivity in the cassette was highly reproducible (Figure 3), indicating the robustness of the production process and Modular-Lab PharmTrace platform performance.

The stability of DO3A-VS-Cys^40^-Tuna-2 was of concern as it contains a tryptophan amino acid residue, which is sensitive to radiolytic oxidation and, thus, might cause formation of radioactive by-products, especially under labeling conditions using a high amount of radioactivity and elevated temperature. The suppression of the radiolysis was achieved by addition of ethanol and dihydroxybenzoic acid as radical scavengers (Figure 4). Ascorbic acid that was essential in the production of [^68^Ga]Ga-DO3A-VS-Cys^40^-Exendin-4 [18] was not necessary, presenting an advantage as, previously, a reduction in radiochemical yield dependent on the concentration of ascorbic acid was observed for [^68^Ga]Ga-DO3A-VS-Cys^40^-Exendin-4 [18]. Thus, the omission of ascorbic acid resulted in the higher radiochemical yield of the crude product in the case of [^68^Ga]Ga-DO3A-VS-Cys^40^-Tuna-2. In general, [^68^Ga]Ga-DO3A-VS-Cys^40^-Tuna-2 was less sensitive to the oxidation than [^68^Ga]Ga-DO3A-VS-Cys^40^-Exendin-4; nevertheless, if, in the case of manual labeling [15], radiolytic oxidation suppression was achieved using only ethanol, the automated labeling needed also dihydroxybenzoic acid. It is worth mentioning that no formation of the oxidized product was detected upon the storage of the aqueous solution of DO3A-VS-Cys^40^-Tuna-2 at −20 °C for at least six months, in contrast to DO3A-VS-Cys^40^-Exendin-4 wherein the oxidation products could be observed within a month.

### 4.2. Quality Control and Clinical Use Aspects

The generator eluate fractionation method used in the labeling synthesis required investigation of the generator elution profile in order to select the top fraction containing over 90% of the maximum possible radioactivity amount. This additional optimization was required in contrast to the synthesis methods wherein the eluate pre-concentration [18] is involved as, in those cases, the only parameter that is critical is the total volume of the eluate, and that can be set to the maximum of 9–10 mL to assure complete radioactivity elution. Other critical parameters that are recommended for investigation are ^68^Ge breakthrough and elution yield, even though the generator is of pharmaceutical grade and, thus, of guaranteed quality. The generator elution 4–20 h prior to the synthesis is recommended in order to keep the competing metal cation impurities at the lowest possible level [21,22].

UV-radio-HPLC used as the major quality control method was thoroughly developed and validated to assure nearly quantitative recovery from the HPLC analytical column and sharp and symmetrical HPLC signals for high accuracy of the signal integration, as well as linearity of the UV-absorption within the range of the expected peptide amount in the quality control sample of the formulated product (Figure 6). UV-HPLC, using calibration plots and one-point system suitability tests, provided accurate quantification of the peptide content and determination of the peptide amount administered to the patients. [^68^Ga]Ga-DO3A-VS-Cys^40^-Tuna-2 was stable at ambient temperature with an RCP of over 95% for at least 2 h (Figure 5); however, due to the requirement for the highest possible molar activity, the radiopharmaceutical was used upon the delivery. The non-decay-corrected radiochemical yield of 45.2 ± 2.5% allowed relatively high molar activity values relevant to the investigated target density. The amount of the administered peptide was less than 30 nmol, adhering to the microdosing guidelines. The quality of the radiopharmaceutical adhered to the pre-defined GMP specifications for safe clinical use (Table 2).

The accurate determination and control of the administered peptide amount were necessary because of the nature of the occupancy study requiring a consistent and relatively low amount of the peptide in the [^68^Ga]Ga-DO3A-VS-Cys^40^-Tuna-2 preparation in order to avoid tissue uptake variability and saturation of the target receptors due to peptide mass effects. At the same time, the radioactivity amount associated with the peptide had to be sufficient for the generation of high-quality images and accurate quantification of the radioactivity uptake in the target tissue. Specific activity (MBq/µg) was determined as the ratio of radioactive material (MBq) to the total content of the peptide (µg) in the formulated preparation. It was used for the estimation of the injected peptide mass (injected mass [µg]) after measurement of the radioactivity in the syringe before and after administration to obtain the injected radioactivity amount (injected radioactivity [MBq]) (Equation (1)).

(1) $$Injected\;mass\;\left[µg\right]=\frac{Injected\;radioactivity\;\left[MBq\right]}{Specific\;activity\;\left[\frac{MBq}{µg}\right]}$$

The dose escalation study conducted previously in non-human primates indicated that the amount of the injected peptide should be less than 0.2 µg/kg body weight in order to minimize the effect of the radiopharmaceutical on the glucagon receptor and enable the assessment of the occupancy at the glucagon receptor by a drug [19]. Thus, it was critical to develop a production method that would allow not only sufficiently high molar activity but also accurate determination of the injected amount of the peptide. The amount of the injected peptide was kept below 0.2 µg/kg body weight (on average, 0.12 ± 0.06 µg/kg, *n* = 21). The corresponding amount of the administered radioactivity (0.47 ± 0.04 MBq/kg body weight, *n* = 21) in combination with PET/CT scanners equipped with digital PET detectors was sufficient to provide high-quality images and accurate quantification. Figure 7 demonstrates a typical baseline distribution of [^68^Ga]Ga-DO3A-VS-Cys^40^-Tuna-2 in a T2D patient with strong liver uptake expected due to the high glucagon receptor expression on hepatocytes.

## 5. Conclusions

Automated and GMP/GRPP-compliant production of [^68^Ga]Ga-DO3A-VS-Cys^40^-Tuna-2 was developed, implemented, and validated on the Modular-Lab PharmTrace synthesis platform. The process was highly reproducible, providing a consistent and accurately determined product peptide concentration relevant for clinical studies of hepatic GCG receptor availability under a microdosing premise.

## Figures and Tables

**Table 1:** Process validation test methods, acceptance criteria, and results for three consecutive productions of [^68^Ga]Ga- DO3A-VS-Cys^40^-Tuna-2 (GCG).

Test Parameter	Test Method	Acceptance Criteria	GCG17008	GCG17009	GCG17010
**Radiochemical purity**	HPLC (retention time)	≥90% no impurity > 5%	98.83	99.88	99.10
**pH**	pH paper	4.0–8.0	6	6	6
**System suitability test (SST)**	HPLC (retention time)	SST corresponds to reference chromatogram	3.69	4.38	4.38
**Identity**	HPLC (retention time)	Agreement between radioactivity and SST UV signals	3.74	4.41	4.42
**Chemical Purity**	HPLC	Absence of unknown UV signals	Absent	Absent	Absent
**Ethanol**	GC chromatography	≤10%	5.1	4.9	4.8
**Filter-Integrity**	Pressure test	* ≥ 1 bar; ** > 3.45 bar	1.75	>3.45	2.0
**Sterility**		Absence of anaerobe or aerobe bacterial growth	Absent	Absent	Absent
**Endotoxins**	LaL	≤0.25 EU/mL	≤0.25 EU/mL	≤0.25 EU/mL	≤0.25 EU/mL
**^68^Ge-Break through**	Radioactivity measurement	<0.001%	0.8 × 10^−5^	0.4 × 10^−5^	0.1 × 10^−5^
**Radionuclide Purity**		>99.9%	>99.9999	>99.9999	>99.999
**Stability**	HPLC	≥90%, no impurity > 5% within 120 min	97.51	98.89	97.26
**Radioactivity Concentration**		5–500 MBq/mL	60	65	62
**Radio Activity**		50–1000 MBq	350	384	350
**Volume**	Syringe or balance	5–7 mL	5.8	5.9	5.64
**Color**	Visual	Colorless	Colorless	Colorless	Colorless

Filter integrity test was performed on * Eckert-Ziegler ModularLab Pharmtracer in-line or on ** standalone device conducting bubble point test.

**Table 2:** Summary of the product specifications and results *.

Test	Acceptance Criteria	[^68^Ga]Ga DO3A-VS-Cys^40^-Tuna-2
Radiochemical purity	>90%; no unknown impurity corresponds to > 5%	99.3 ± 0.5
pH	4–8.5	6.0
Radioactivity concentration	5–100 MBq/mL	62.5 ± 2.4
Radioactivity	50–500 MBq	361 ± 20
Volume	2–10 mL	5.78 ± 0.13
Color	colorless	colorless
Radionuclidic purity	>99.9%	99.99999 ± 0.000006
^68^Ge breakthrough	<0.001%	0.000043 ± 0.000004
Stability	RCP ** >91% within 120 min	97.9 ± 0.9
Ethanol content	<10%	4.9 ± 0.2

* The results are presented as mean ± SD (*n* = 3, process validation runs). ** Radiochemical purity.

**Table 3:** Acceptance criteria and results of the UV-radio-HPLC analysis method validation.

Description	Acceptance Criteria	Result
Linearity—UV detector Pearson correlation coefficient (*R^2^*)	>0.99	>0.999
Precision as repeatability—UV (Relative standard deviation)	≤5%	4 (*n* = 6)
Specificity—Radio detector (Relative standard deviation)	≤5%	1.0 (*n* = 6)
Column recovery ([^68^Ga]Ga- DO3A-VS-Cys^40^-Tuna-2 spiked with [^68^Ga]GaCl_3_)	≥95%	>97
Column recovery ([^68^Ga]Ga- DO3A-VS-Cys^40^-Tuna-2)	≥95%	>99
